# Association of social vulnerability index and masking adherence among children enrolled in COVID-19 community research partnership study

**DOI:** 10.1186/s12889-024-17931-1

**Published:** 2024-02-08

**Authors:** Keerti L. Dantuluri, Asare Buahin, Diane Uschner, Michael E. DeWitt, Whitney Rossman, Connell O. Dunn, Timothy C. Hetherington, Jennifer Priem, Paola Castri, William H. Lagarde, Michael Gibbs, Amina Ahmed, The COVID-19 Community Research Partnership

**Affiliations:** 1 Department of Pediatrics (Infectious Diseases) at Levine Children’s Hospital and Wake Forest University School of Medicine, Atrium Health, 1001 Blythe Blvd, Medical Education Building, P.O. Box 32861, Charlotte, NC 28203 USA; 2 Department of Biostatistics and Bioinformatics and The Biostatistics Center at The George Washington University, Washington, D.C., USA; 3 Section on Infectious Diseases, Department of Medicine at Wake Forest University School of Medicine and the Department of Biology, Wake Forest University, Winston-Salem, NC USA; 4 Center for Outcomes Research and Evaluation at Atrium Health, Charlotte, NC USA; 5 Department of Emergency Medicine Research at Atrium Health, Charlotte, NC USA; 6 Department of Neurology (Pediatric Neurology) at Wake Forest Baptist, Winston Salem, NC USA; 7 Department of Pediatrics (Endocrinology) at WakeMed, Raleigh, NC USA; 8 Department of Emergency Medicine at Atrium Health, Charlotte, NC USA

**Keywords:** COVID-19, Social vulnerability index, Pediatrics, Masking, Infection prevention, Public health

## Abstract

**Background:**

Individuals with high social vulnerability index (SVI) have poorer outcomes with COVID-19. Masking reduces transmission of COVID-19 among children, but how SVI plays a role in masking behavior is unknown. We aimed to measure the association of SVI with masking adherence among children during the COVID-19 pandemic.

**Methods:**

We conducted a multi-site, prospective syndromic surveillance study among children aged 2 – 17 years in the Southeastern United States by daily electronic surveys which solicited symptoms of COVID-19-like illness, infection with or exposure to SARS-CoV-2, masking habits, and any receipt of COVID-19 vaccines. Parents/guardians submitted surveys for their children; adolescents 13 years and older could opt to submit their own surveys. Multivariable and univariate linear models were used to measure the associations of different predictors such as SVI with masking adherence.

**Results:**

One thousand four hundred sixty-one children from 6 states and 55 counties predominately from North and South Carolina were included in the analysis. Most children in the cohort were 5 – 11 years old, non-Hispanic White, from urban counties, and with low-moderate SVI. Overall masking adherence decreased over time, and older children had higher masking adherence throughout the study period compared with younger children. Children who resided in urban counties had greater masking adherence throughout the study period than those who resided in suburban or rural counties. Masking adherence was higher among children with both low and medium SVI than those with high SVI.

**Conclusions:**

Despite being at risk for more severe outcomes with COVID-19, children with high SVI had lower levels of masking adherence compared to those with low SVI. Our findings highlight opportunities for improved and targeted messaging in these vulnerable communities.

## Background

During the COVID-19 pandemic, the Centers for Disease Control and Prevention (CDC) and World Health Organization recommended masking to mitigate the transmission of SARS-CoV-2 infection [1, 2]. Mask mandates are associated with a decrease in spread of infection at both local and state level [3]. Despite evidence supporting masking, mandating masks among children, particularly in schools, is controversial [4]. While the prevalence of masking mandates has decreased as the pandemic has evolved, masking is still a measure that vulnerable populations can take to reduce their risk of infection.

Several studies have demonstrated that individuals with high social vulnerability index (SVI), which leverages United States Census data to describe social vulnerability of counties and tracts based on social factors such as poverty, housing, and access to transportation, are at greater risk for increased morbidity and mortality with COVID-19 [5–7]. Racial and ethnic disparities in the severity of COVID-19 and multisystem inflammatory syndrome in children (MIS-C) are well established [8, 9]. Immunization, the other major mechanism to mitigate acquisition and transmission of SARS-CoV-2 infection, is also variable based upon SVI; adults and children from counties with higher SVIs are less likely to be immunized against COVID-19 [10]. Barriers to immunizations among families with higher SVI include health literacy and financial and transportation limitations. However, the association of SVI and masking behavior, particularly among children, is less clear.

Because of the known disparities of COVD-19 outcomes in children with high SVI, it is critical for public health officials to understand the impact of risk-reducing interventions, like immunization and masking. Factors associated with masking are more multi-faceted than those associated with immunization. Psychosocial factors such as racial and ethnic identity, community norms, and political connotations of masking may play a role in masking behavior [11, 12]. Understanding patterns in masking behavior among children during a pandemic can help public health officials allocate resources and target educational interventions in high-risk areas with lower masking prevalence. We measured the prevalence of masking adherence among children enrolled in a syndromic surveillance study in the Southeastern United States.

## Methods

The COVID-19 Community Research Partnership (CCRP) is a multi-site, prospective study combining electronic symptom surveillance with at-home longitudinal serological and virological surveillance in adults and children [13, 14]. The pediatric arm of the study included children 2 – 17 years of age. Children were enrolled through large healthcare systems serving populations predominantly in North and South Carolina, from April 2 through June 24, 2021. The study was approved by a centralized Institutional Review Board. Participants were recruited through email, public-facing advertisement, and in-person engagements. Community-based partnerships were used to recruit minorities at community events, through churches, and ethnic grocery stores. During enrollment, participants or parents/guardians of participants provided informed consent to symptom surveillance alone or symptom surveillance and at-home serological and virological testing; adolescents 13 years and older provided assent. Participants self-reported demographic data and health history including prior history of SARS CoV-2 infection at enrollment. Daily electronic surveys developed and administered by Oracle Corporation (Redwood, CA, USA) solicited symptoms of COVID-19-like illness, infection with or exposure to SARS-CoV-2, masking habits, and any receipt of COVID-19 vaccines. Parents/guardians submitted surveys for and conducted at-home tests on their children; adolescents 13 years and older could opt to submit their own surveys. Further details of the study protocol were previously published [15]. Syndromic surveillance was completed on December 31, 2021.

We included in the analysis participants who submitted surveys consistently, defined as survey submission at least once per week for at least 12 weeks consecutively. Participants’ home addresses were securely geocoded and associated with a census tract and corresponding CDC SVI [16]. If a child lived with more than one caregiver with different residences, we used the address of the caregiver who enrolled the child, with the stipulation that the child lives at least part-time with this caregiver. The CDC SVI data included the overall SVI score as well as RPL_THEMES, which is an overall tract summary ranking variable of four themes related to social vulnerability: socioeconomic status, household composition and disability, minority status and language, and housing type and transportation. Participants from census tracts that did not have an associated RPL_THEMES value were excluded from the analysis. Participant SVI data were categorized into tertiles of nearly equal number of observations.

Masking adherence was defined as wearing a mask when interacting with people outside of the household. We used a binary scale of adherent or non-adherent to masking. Adherence to masking was captured by participants responding in the survey that they masked “all of the time” or “some of the time” if they “interacted closely (within 6 feet) with people outside of [their] immediate household.” Adherence was calculated as the proportion of time during survey enrollment that participants wore a mask when interacting with people outside of their household. The primary outcome of interest was the reported masking adherence over time and the associations of race/ethnicity, age, sex, rurality of residence, and SVI. Rurality was determined by the participant’s county of residence, which was defined as rural, suburban, or urban, based upon population density [15, 17]. Summary statistics were completed to describe the study cohort. Multivariable and univariate linear models were used to explore the associations of different predictors with masking adherence. Univariate analysis was conducted for each of the above variables. Multivariable models were fit with univariate predictors with statistical significance defined as a *p*-value ≤ 0.05. All analyses were conducted in Python version 3.8 and R version 4.0.1.

## Results

A total of 3,310 pediatric participants were enrolled in the study. Of those, 12 were excluded because of missing RPL_THEMES data. Of the remaining 3,298 participants, 1,873 participants were excluded due to not answering the survey for at least 12 consecutive weeks. Therefore, a total of 1,461 pediatric participants met inclusion criteria for analysis, with the mean participant reporting 4.9 times per week and the median participant reporting 5 times per week. Participants came from 6 states and 55 counties. The majority of children were from North Carolina (*n* = 1,296, 88.7%) and South Carolina (*n* = 158, 10.8%), with lower representation from Virginia (*n* = 4, 0.3%) and one participant each from Florida, Illinois, and Mayland. Most children were 5 – 11 years of age (47%), non-Hispanic White (80.2%), and from urban counties (54.7%) (Table 1). More than 70% of the cohort had a SVI value between 0.0007 and 0.441, reflecting low-moderate social vulnerability (Table 1).

**Table 1:** Characteristic of pediatric participants in syndromic surveillance

	**Overall (*N* = 1461)**
**Sex**	
Female	766 (52.4%)
Male	695 (47.6%)
**Age (years)**	
2–4	259 (17.7%)
5–11	686 (47.0%)
12–17	516 (35.3%)
**Race/Ethnicity**	
Non-Hispanic White	1171 (80.2%)
Non-Hispanic Other	129 (8.8%)
Non-Hispanic Black	85 (5.8%)
Hispanic	76 (5.2%)
**Rurality of County of Residence**	
Urban	799 (54.7%)
Suburban	483 (33.1%)
Rural	179 (12.3%)
**Social Vulnerability**	
Low^a^	557 (38.1%)
Medium^b^	483 (33.1%)
High^c^	421 (28.8%)

^a^Low: SVI in range (0.0007, 0.2]

^b^Medium: SVI in range (0.2, 0.44]

^c^High: SVI in range (0.44, 0.984]

**Fig. 1:**
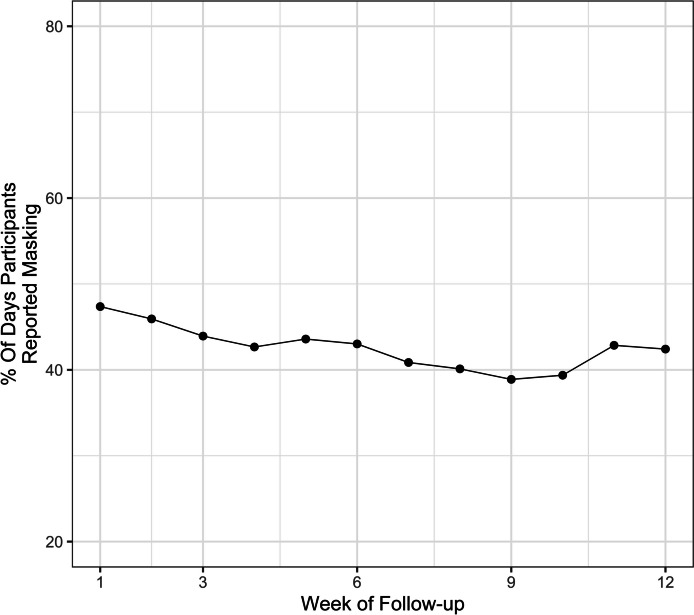
Overall masking adherence

**Fig. 2:**
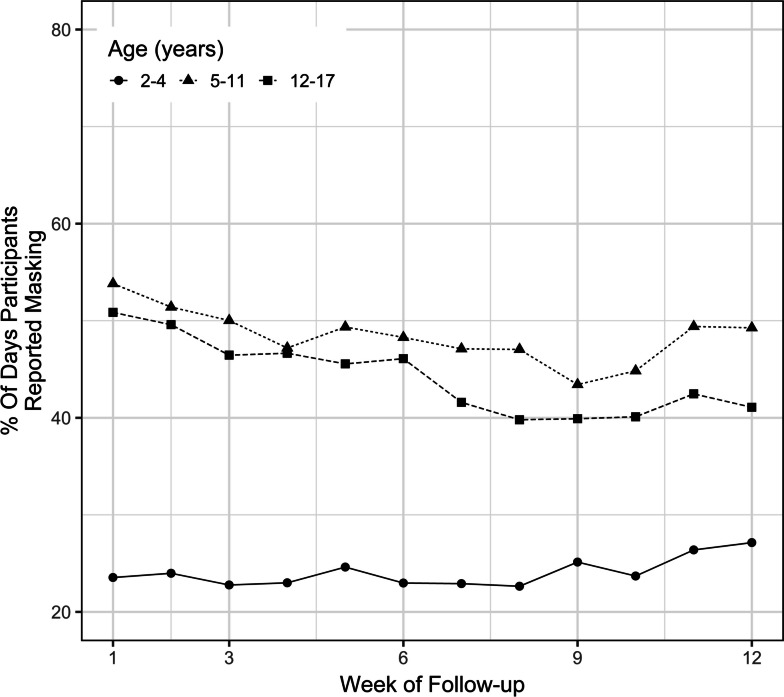
Masking adherence by age

**Fig. 3:**
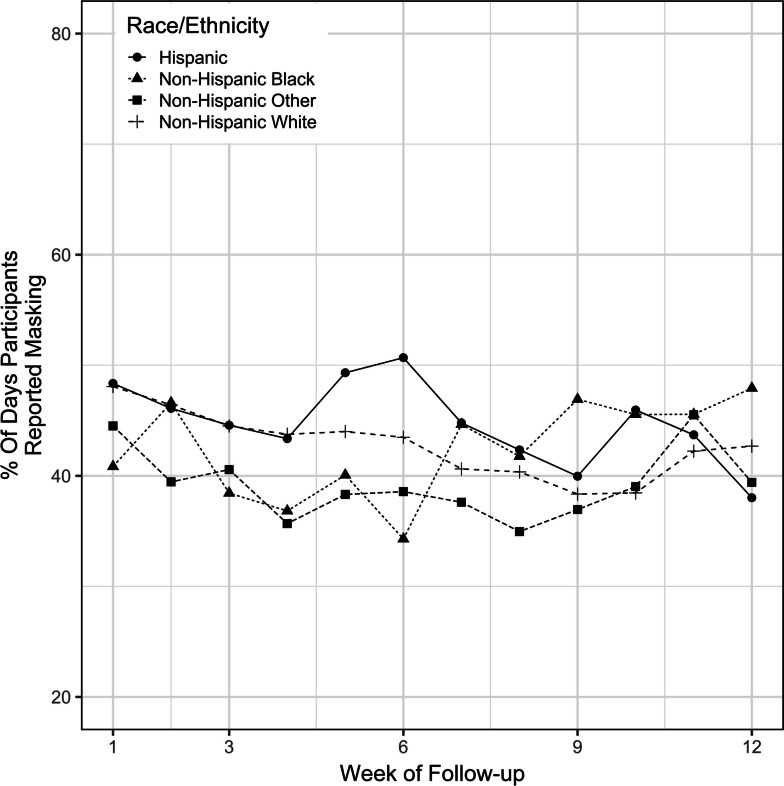
Masking adherence by race/ethnicity

**Fig. 4:**
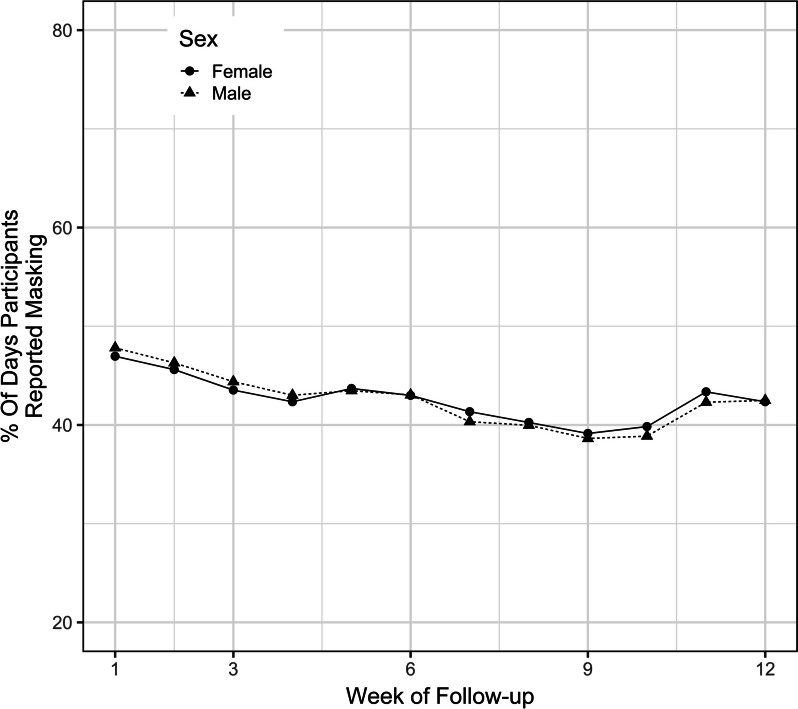
Masking adherence by sex

The overall percentage of participants who were adherent to masking started at a maximum of nearly 50% at the beginning of the study and then decreased to a minimum of 39% at week 10 and ended at 43% at the end of the study (Fig. 1). Children aged 5 – 17 years had higher masking adherence throughout the study compared to children aged 2 – 4 (Fig. 2). The univariate analysis revealed a 1.04% (95% confidence interval [CI] 0.72, 1.36) increase in masking adherence with each year of increase in age. The multivariable analysis, which adjusted for race, ethnicity, sex, rurality of residence, and SVI, revealed a 1.07% (95% CI 0.76, 1.39) increase in masking adherence with each year of increase in age. Masking adherence was not statistically different among different racial/ethnic groups or between male and female sex (Figs. 3 and 4).

**Fig. 5:**
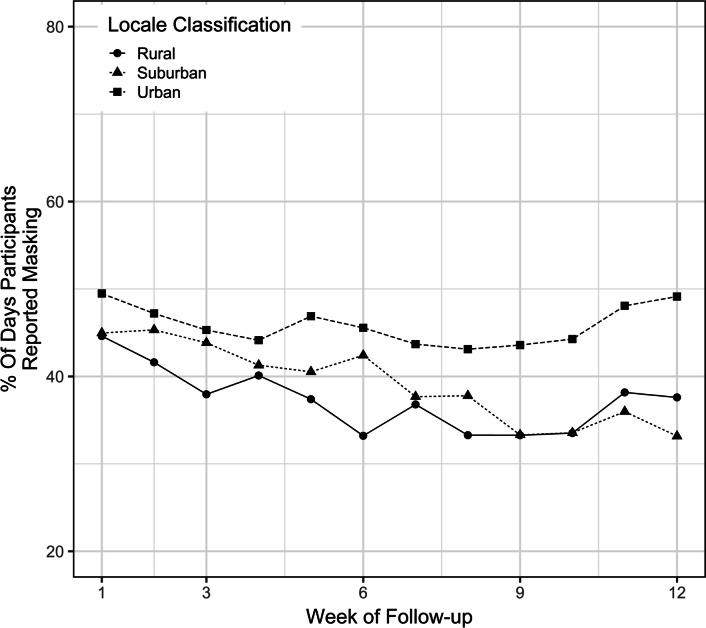
Masking adherence by rurality of county of residence

**Fig. 6:**
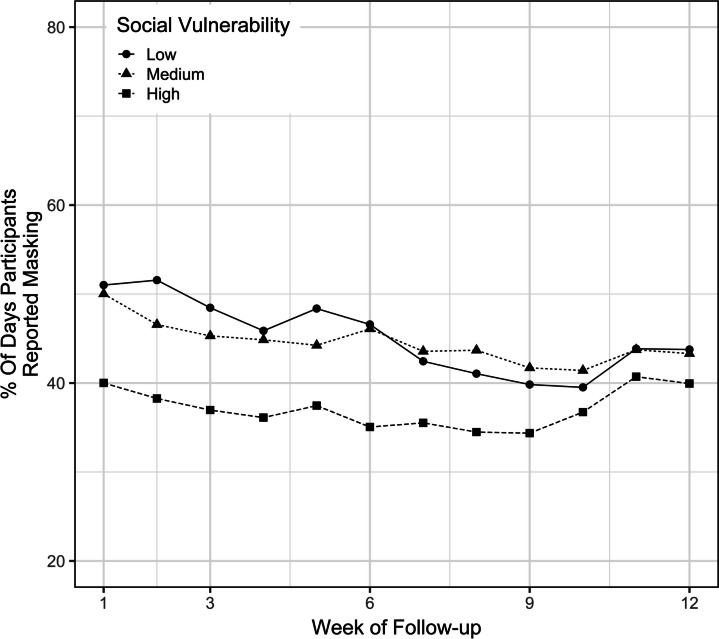
Masking adherence by social vulnerability index (SVI)

Children who resided in urban counties had greater masking adherence throughout the study than those who resided in suburban or rural counties, with a difference of 6.70% (CI 3.68, 9.72) and 8.55% (CI 4.22, 12.9) by univariate analysis, respectively, and a difference of 7.08% (CI 4.08, 10.1) and 5.60% (CI 0.66, 10.5) by multivariable analysis, respectively (Fig. 5). Although there was no difference detected in masking adherence between children with low and medium SVI, the masking adherence was higher among children with either low or medium SVI than those with high SVI (Fig. 6). Children with the lowest SVI had 8.06% (CI 4.56, 11.6) and 6.09% (CI 2.23, 9.96) higher masking adherence than those with the highest SVI by univariate and multivariate analyses, respectively.

## Discussion

In our multi-site, prospective CCRP study, we measured patterns associated with masking adherence among children during the COVID-19 pandemic. Overall masking adherence decreased slightly across the study period. We suspect this was due to public messaging on masking guidance changing over time as well as pandemic fatigue, which refers to the decline in adherence to infection prevention guidelines secondary to pandemic-related emotional burnout [18, 19]. Masking adherence was also lower among the youngest age group, who may be more behaviorally reticent to wear masks or need more guidance from adults to readjust their masks [20]. Children who resided in rural and suburban counties exhibited lower masking adherence than those who resided in urban settings, a finding that is supported by previously published studies [21, 22]. Our study adds to the literature by demonstrating masking differences among children based upon their SVI. Children in our cohort with the highest SVI had the lowest masking adherence.

Prior studies have established that social factors can contribute negatively to health outcomes. Higher SVI is associated with greater risks of childhood obesity, asthma exacerbations, and cardiovascular disease [23–25]. During the pandemic, similar associations were found between SVI and COVID-19 outcomes. One ecological study measuring the effect of SVI on the incidence of COVID-19 in Louisiana between March and August 2020 found a 52% higher risk of SARS-CoV-2 infection in census tracts with higher levels of SVI even after adjusting for population density [5]. In addition to greater risk of infection, higher SVI was also associated with greater risk of mortality from COVID-19 [6]. To assess severity of outcomes in children, another study utilized data from the Overcoming COVID-19 registry and found that children with high SVI had 2.03 times greater odds of developing MIS-C than those with low SVI, and those with moderate SVI had 1.88 greater odds of developing MIS-C than those with low SVI [26]. Although Black and Hispanic children have higher SVI and higher rates of MIS-C, SVI was associated with a greater likelihood of MIS-C even after adjusting for racial, ethnic, and other demographic factors [26]. This association of SVI and severe outcomes from COVID-19 highlights the role socioeconomic stress may play in the dysregulation of the immune system’s response to SARS-CoV-2. Individuals from lower socioeconomic status often have barriers to food security, shelter, and access to health care (particularly preventative health care), which can all negatively impact the clinical outcomes after infection.

The association of high SVI with poor COVID-19 outcomes indicates the need to better understand and optimize infection prevention interventions in this vulnerable pediatric population. Early in the pandemic, adults with higher SVI were less able to adhere to stay-at-home recommendations [27]. These individuals are more likely to have essential jobs without work-from-home options and fewer resources to sustain a household without working. In turn, children from the same households as these caregivers were also at increased risk for acquiring infection. Because factors outside of their control can prevent individuals with high SVI from adhering to social distancing precautions, efforts to prevent transmission of infection should be focused on immunization and masking in this population.

Our study findings of lower masking adherence among children with higher SVI echo vaccine uptake within these communities. Several studies have demonstrated lower COVID-19 vaccine uptake among individuals with higher SVI during the pandemic [28]. Unfortunately, efforts to increase vaccination sites in settings with high vulnerability populations have not mitigated this disparity in immunization against COVID-19 [28]. Pediatric-specific efforts to increase vaccine uptake were also associated with persistent disparities based upon SVI. After 11 weeks of implementing a national pediatric vaccine program on November 1, 2021, 54% of providers were established in high SVI areas, but the two dose vaccine series was completed by only 13.7% of the population in these high SVI areas, compared to 21.7% in low SVI areas [29]. Reasons for this disparity include parental vaccine hesitancy and discordance in messaging regarding vaccine information in the community. In addition to improving messaging by collaborating with trusted stakeholders in communities to address parental concerns regarding immunization, addressing disparities in masking adherence is also vital to further reduce the burden of infection in high-risk communities.

Our findings of lower rates of masking among children with higher SVI is crucial as this infection prevention measure is being underutilized in the population most at risk for severe outcomes from COVID-19. Although immunization decreases the risk of severe outcomes of COVID-19, including MIS-C, improving masking adherence can reduce SARS-CoV-2 transmission. A prospective nested case–control CCRP study in adults revealed that lack of masking adherence was associated with 49% higher odds of acquiring SARS-CoV-2 infection during November 2020 – October 2021 than consistently masking [30]. This association persisted despite participant immunization status, underscoring the additive value of masking during periods with high risk of infection transmission.

Lower rates of masking among children with higher SVI highlights the need to improve messaging and allocation of resources in their communities. One qualitative study which utilized a focus group of North Carolina residents to understand motivations and barriers to masking demonstrated that the desire to protect oneself and others against infection was a key driver in masking adherence [31]. Another focus group study from Canada found that despite their desire to adhere to guidelines backed by scientific evidence, inconsistent public health messaging and lack of clear rationales behind masking caused confusion and mistrust towards healthcare professionals, leading to reduction in masking adherence [32]. Developing focus groups with parents and caregivers from predominantly high SVI regions can be targeted for future studies to determine how to tailor messaging to improve masking adherence in children from this vulnerable population. Furthermore, ensuring high SVI regions have abundant access to high-quality masks can also assist in optimizing masking adherence and reduction in transmission of infection [33].

Our study addresses a gap in the literature regarding the relationship between masking behavior and SVI, especially in children. Strengths of the study include a large sample size covering a wide region in the Southeast United States, which ranks lower than the rest of the country in healthcare status and outcomes among individuals [34]. The prospective nature of our surveillance study also allowed us to assess changes in individual masking patterns over time as the pandemic evolved.

Limitations of our study include the use of calendar time for trending changes in masking adherence. Because heterogeneity exists in the start and stop dates for follow-up of participants in our study, the role of masking policies in masking adherence cannot be incorporated into the analysis. However, in general, our study took place during the Delta variant phase, when the CDC recommended that everyone, regardless of their vaccination status, wear a mask indoors in areas with high prevalence of COVID-19. Because our study is limited to the Delta variant phase, we did not address masking adherence during the Omicron variant phase, which was associated with increased transmissibility of the virus. Additionally, we classified our high SVI population as children from the tertile range of 0.4 – 0.8, which is a less granular approach compared to the quartile ranges utilized by the CDC, which defines high SVI as > 0.75 and moderately high SVI as 0.5 – 0.75 [35]. Our inclusion criteria of only individuals who consistently responded to the survey also adds an element of selection bias to our study, as those who responded may have a higher degree of masking compliance (i.e., be more engaged in following policies and public health measures). Additionally, our definition of masking adherence allows a level of subjectivity from the survey respondent as individual thresholds for “some of the time” likely vary among participants. Finally, our findings cannot be generalized to regions outside of North and South Carolina or communities with a high density of minority populations. Although we enhanced recruitment efforts of minority children, our study still underrepresented minority children, including those with higher SVI [36]. Future studies should aim to enrich the enrollment of minority children to better understand masking patterns in this group. Additionally, further qualitative studies to better understand the reasons behind masking adherence would be helpful to inform interventions to improve masking rates among vulnerable populations.

## Conclusions

Children with higher SVI have lower masking adherence and should be the target of public health interventions to improve the uptake of infection prevention measures in populations at greatest risk for infection and poor outcomes secondary to infection. Ongoing surveillance of infection, vaccination, and masking patterns would be helpful to continue protecting the most vulnerable populations in the post-pandemic era.

## Data Availability

The datasets used and/or analyzed during the current study are available from the corresponding author upon reasonable request.

## Abbreviations

CDC: Centers for Disease Control and Prevention
SVI: Social Vulnerability Index
MIS-C: Multisystem Inflammatory Syndrome in Children
CCRP: COVID-19 Community Research Partnership

## References

[1] Centers for Disease Control and Prevention. Use and Care of Masks. https://www.cdc.gov/coronavirus/2019-ncov/prevent-getting-sick/about-face-coverings.html. Published February 25, 2022. Accessed 5 Aug 2022.

[2] World Health Organization. Coronavirus disease advice for the public: when and how to use masks. https://www.who.int/emergencies/diseases/novel-coronavirus-2019/advice-for-public/when-and-how-to-use-masks. Published December 2021. Accessed 5 Aug 2022.

[3] Adjodah D, Dinakar K, Chinazzi M (2021). Association between COVID-19 outcomes and mask mandates, adherence, and attitudes. Capraro V, ed. PLoS One.

[4] Spitzer M (2020). Masked education? The benefits and burdens of wearing face masks in schools during the current Corona pandemic. Trends Neurosci Educ.

[5] Biggs EN, Maloney PM, Rung AL, Peters ES, Robinson WT (2020). The relationship between social vulnerability and COVID-19 incidence among Louisiana census tracts. Front Public Health.

[6] Freese KE, Vega A, Lawrence JJ, Documet PI (2021). Social vulnerability is associated with risk of COVID-19 related mortality in U.S. counties with confirmed cases. J Health Care Poor Underserved.

[7] Islam SJ, Nayak A, Hu Y (2021). Temporal trends in the association of social vulnerability and race/ethnicity with county-level COVID-19 incidence and outcomes in the USA: an ecological analysis. BMJ Open.

[8] Saatci D, Ranger TA, Garriga C (2021). Association between race and COVID-19 outcomes among 2.6 million children in England. JAMA Pediatr.

[9] Stierman B, Abrams JY, Godfred-Cato SE (2021). Racial and ethnic disparities in multisystem inflammatory syndrome in children in the United States, March 2020 to February 2021. Pediatr Infect Dis J.

[10] Barry V, Dasgupta S, Weller DL (2021). Patterns in COVID-19 vaccination coverage, by social vulnerability and urbanicity - United States, December 14, 2020-May 1, 2021. MMWR Morb Mortal Wkly Rep.

[11] Kahn KB, Money EEL (2022). (Un)masking threat: racial minorities experience race-based social identity threat wearing face masks during COVID-19. Group Process Intergroup Relat.

[12] Cunningham GB, Nite C (2021). Demographics, politics, and health factors predict mask wearing during the COVID-19 pandemic: a cross-sectional study. BMC Public Health.

[13] Herrington DM, Sanders JW, The COVID-19 Community Research Partnership Study Group (2021). Duration of SARS-CoV-2 sero-positivity in a large longitudinal sero-surveillance cohort: the COVID-19 Community Research Partnership. BMC Infect Dis.

[14] The COVID-19 Community Research Partnership, Sanders J. The COVID-19 community research partnership: objectives, study design, baseline recruitment, and retention. Infectious Diseases (except HIV/AIDS); 2022. 10.1101/2022.02.09.22270272.

[15] COVID-19 Community Research Partnership. The COVID-19 Community Research Partnership: a multistate surveillance platform for characterizing the epidemiology of the SARS-CoV-2 pandemic. Biol Methods Protoc. 2022;7(1):bpac033. 10.1093/biomethods/bpac033. 10.1093/biomethods/bpac033 PMC9789889 36589317

[16] Centers for Disease Control and Prevention. CDC SVI 2018 Documentation. https://svi.cdc.gov/Documents/Data/2018_SVI_Data/SVI2018Documentation.pdf. Published January 31, 2020.

[17] United States Census Bureau. American Community Survey Data Profiles. Census.gov. https://www.census.gov/programs-surveys/acs/. Accessed 7 June 2023.

[18] Rogers K, Fandos N. Removing masks becomes the first bipartisan activity of Biden’s Washington. The New York Times. https://www.nytimes.com/2021/05/13/us/politics/washington-mask-guidance.html. Published May 13, 2021. Accessed 29 June 2023.

[19] Taylor S, Rachor GS, Asmundson GJG (2022). Who develops pandemic fatigue? Insights from latent class analysis. PLoS One.

[20] Mickells GE, Figueroa J, West KW, Wood A, McElhanon BO (2021). Adherence to masking requirement during the COVID-19 pandemic by early elementary school children. J Sch Health.

[21] Callaghan T, Lueck JA, Trujillo KL, Ferdinand AO (2021). Rural and urban differences in COVID-19 prevention behaviors. J Rural Health.

[22] Pro G, Schumacher K, Hubach R (2021). US trends in mask wearing during the COVID-19 pandemic depend on rurality. Rural Remote Health.

[23] Aris IM, Perng W, Dabelea D (2022). Associations of neighborhood opportunity and social vulnerability with trajectories of childhood body mass index and obesity among US children. JAMA Netw Open.

[24] Nayak SS, Borkar R, Ghozy S (2022). Social vulnerability, medical care access and asthma related emergency department visits and hospitalization: an observational study. Heart Lung.

[25] Jain V, Al Rifai M, Khan SU (2022). Association between social vulnerability index and cardiovascular disease: a behavioral risk factor surveillance system study. J Am Heart Assoc.

[26] Zambrano LD, Ly KN, Link-Gelles R (2022). Investigating health disparities associated with multisystem inflammatory syndrome in children after SARS-CoV-2 infection. Pediatr Infect Dis J.

[27] Fletcher KM, Espey J, Grossman MK (2021). Social vulnerability and county stay-at-home behavior during COVID-19 stay-at-home orders, United States, April 7–April 20, 2020. Ann Epidemiol.

[28] Thakore N, Khazanchi R, Orav EJ, Ganguli I (2021). Association of social vulnerability, COVID-19 vaccine site density, and vaccination rates in the United States. Healthc (Amst).

[29] Kim C, Yee R, Bhatkoti R (2022). COVID-19 vaccine provider access and vaccination coverage among children aged 5–11 years - United States, November 2021-January 2022. MMWR Morb Mortal Wkly Rep.

[30] Tjaden AH, Edelstein SL, Ahmed N (2023). Association between COVID-19 and consistent mask wearing during contact with others outside the household—a nested case–control analysis, November 2020–October 2021. Influenza Resp Viruses.

[31] Shelus VS, Frank SC, Lazard AJ (2020). Motivations and barriers for the use of face coverings during the COVID-19 pandemic: messaging insights from focus groups. Int J Environ Res Public Health.

[32] Zhang YSD, Young Leslie H, Sharafaddin-zadeh Y, Noels K, Lou NM (2021). Public health messages about face masks early in the COVID-19 pandemic: perceptions of and impacts on Canadians. J Community Health.

[33] Worby CJ, Chang HH (2020). Face mask use in the general population and optimal resource allocation during the COVID-19 pandemic. Nat Commun.

[34] America’s Health Rankings. 2017 Annual Report. America’s Health Rankings. https://www.americashealthrankings.org/learn/reports/2017-annual-report. Accessed 14 June 2023.

[35] CDC/ATSDR SVI Frequently Asked Questions (FAQ) | Place and Health | ATSDR. https://www.atsdr.cdc.gov/placeandhealth/svi/faq_svi.html. Published October 26, 2022. Accessed 29 June 2023.

[36] Dantuluri KL, Rossman W, Lu LC, et al. Improving minority representation through COVID-19 community research partnership. Infectious Diseases Diagnosis & Treatment. https://www.gavinpublishers.com/article/view/improving-minority-representation-through-covid-19-community-research-partnership. Published online January 12, 2023. Accessed 14 June 2023.

